# Association between social supports and negative emotions among pediatric residents in China: The chain-mediating role of psychological resilience and burnout

**DOI:** 10.3389/fpubh.2022.962259

**Published:** 2023-01-23

**Authors:** Chao Song, Xiao-Tian Du, Yun-Xia Hong, Jian-Hua Mao, Wen Zhang

**Affiliations:** ^1^The Children's Hospital, Zhejiang University School of Medicine, National Clinical Research Centre for Child Health, Hangzhou, China; ^2^School of Public Health, Shanghai Medical College, Fudan University, Shanghai, China; ^3^Department of Philosophy, Beijing Normal University, Beijing, China

**Keywords:** social support, psychological resilience, stress, anxiety, depression, burnout, chain-mediating effect, pediatrician

## Abstract

**Background:**

Chinese pediatricians are facing challenges, and there is a need to examine the issue of negative emotions, namely, stress, anxiety and depression, among front-line pediatric residents in clinical settings. Understanding the current situation and influencing factors of negative emotions among pediatric residents in China and exploring the formation mechanism can lay a foundation for psychological interventions.

**Methods:**

A total of 138 pediatric residents in the Children's Hospital, Zhejiang University School of Medicine, China, were surveyed using the Depression Anxiety Stress Scale-21 (DASS-21), Social Support Rating Scale (SSRS), Connor-Davidson Resilience Scale (CD-RISC), and Maslach Burnout Inventory-General Survey (MBI-GS).

**Results:**

(1) The incidence of abnormal stress, anxiety, and depression among pediatric residents was 18.8%, 47.8%, and 47.8% respectively. (2) Negative emotions were significantly negatively correlated with social supports and psychological resilience, and positively correlated with burnout. (3) The chain-mediating effect of resilience and burnout between social supports and negative emotions was significant.

**Conclusion:**

Psychological resilience and burnout played a chain-mediating role between social supports and negative emotions. Measures should be taken to improve the mental health of Chinese pediatric residents.

## 1. Introduction

In recent years, pediatricians in China have been confronted with a crisis with regards to long working hours, the frequency of medical disputes, and a shortage of pediatricians, which has caused widespread concern among the public. Despite intense workloads and high levels of risk, they earn less income than other senior healthcare providers (1, 2). The turnover rate of pediatricians in China is as high as 12.6%, and largely includes young doctors (3). In China, the standardized training system for residents has been established in recent years. All clinicians, including pediatricians, should undergo a 3-year residency training after graduating from medical school. During these 3 years, residents will study in different departments of the training base in turn. Pediatric residents work on the front line in healthcare settings, have direct interaction with children and their families, and are close to graduating from medical school and embarking on their careers in clinical practice. It is necessary to pay attention to their negative emotions caused by above crisis, because addressing negative emotions is not only the key to improving quality of life (4), but also patient safety (5). Anxiety and depression are the most common negative emotions in the general population (6). Anxiety and depression are usually external manifestations of stress (7). Therefore, we proposed the following hypotheses and verified the mechanisms.

Social supports describe the actual or perceived material, emotional and spiritual supports that an individual can access in their external world (8). The social support buffering hypothesis holds that social supports can mitigate the negative impact of stress on mental health (9). Lower levels of social support have been found to be associated with more mental health issues (10, 11), and individuals with less social support are about five times more likely to experience symptoms of anxiety and depression than those that have greater social supports (12). People with low levels of social support are at a higher risk of psychological stress, while higher levels of social support can help to alleviate anxiety and depression (13–15). Accordingly, the first hypothesis (H1) is proposed: social support can predict negative emotions among pediatric residents.

Psychological resilience is the ability to flexibly deal with challenges and overcome adversity, and it is accompanied by personal growth and transformation (16, 17). Some studies have pointed out that there is a significant positive correlation between social support and psychological resilience (18, 19), and people who receive more social support tend to have a higher level of psychological resilience. Psychological resilience can negatively predict anxiety (20) and alleviate depressive symptoms (21). High levels of resilience are associated with optimism and tenacity (22). Thus, the second hypothesis (H2) is proposed: among pediatric residents, social support could influence negative emotions through the mediating effect of psychological resilience.

Burnout is a psychological condition or a response to chronic emotional and interpersonal stressors on the job (23), and burnout among pediatricians may pose a danger to both doctors and patients (24). Negative emotions are closely related to job burnout (25, 26). Previous study has shown that social support has a negative predictive effect on job burnout among nurses (27). Support from leaders and colleagues could alleviate burnout and contribute to lower turnover intentions (28). Studies in different environments (29) and in different populations (30) seem to have found that social support may have a positive effect on burnout. Accordingly, the third hypothesis (H3) is proposed: among pediatric residents, social support can influence negative emotions through the mediating effect of burnout.

High psychological resilience often enables individuals to cope more effectively with their work environment, improve their work involvement and alleviate symptoms of burnout (31). Resilience can be regarded as the other side of the burnout coin (32) and could protect people from workplace stress (33). Researchers who have examined the relationship between resilience and burnout among nurses (34, 35) have shown that these two factors are negatively correlated, suggesting that higher levels of resilience might protect nurses from emotional exhaustion and contribute to personal accomplishment. Combining the relationship between resilience and social support, and the relationship between burnout and negative emotions, the fourth hypothesis (H4) is put forward: among pediatric residents, social support can affect negative emotions through the chain-mediating effect of resilience and burnout.

By verifying the above hypotheses, it would help exploring the psychological intervention for Chinese pediatric residents to improve their mental health, and reduce the turnover rate of pediatric professionals.

## 2. Methods

### 2.1. Investigation site and participants

The study was conducted at the Children's Hospital, Zhejiang University School of Medicine, Zhejiang Province, Eastern China in January 2022. A total of 138 pediatric residents participated in the investigation. This study was approved by the Ethics Committee of the children's hospital, Zhejiang University school of medicine (No. 2022-IRB-107).

### 2.2. Questionnaire tool

#### 2.2.1. Socio-demographic information

The socio-demographic information questionnaire covered information related to gender, age, working years, education level, annual income, category, qualification, and clinical practice area.

#### 2.2.2. Depression Anxiety Stress Scale-21

The Depression Anxiety Stress Scale-21 (DASS-21) is used to measure an individual's negative mood and the severity of symptoms in the previous week (36). The scale includes three subscales of anxiety, depression, and stress. Each subscale contains seven items, and a total of 21 items. Four grades ranging from 0 to 3 were used: “completely inconsistent,” “partially consistent,” “mostly consistent,” and “completely consistent.” Higher scores indicated more intense negative emotions. Each subscale is divided into *asymptomatic, mild, moderate, severe*, and *extremely severe* according to the score. In this study, the overall Cronbach's (37) alpha value was 0.956, and the Cronbach's alpha coefficients of each subscale were 0.880, 0.879, and 0.892, respectively.

#### 2.2.3. Social Support Rating Scale

The Social Support Rating Scale (SSRS) (38) compiled by Chinese scholar Shuiyuan Xiao consists of ten items and three dimensions: objective social support (four items), subjective social support (four items), and utilization of social support (three items). Items 1–5 and 8–10 were scored on a four-point Likert scale, ranging from 1 (“not at all”) to 4 (“very much”). For items 6 and 7, the response “no source” was assigned 0 points and the response “have a source” was assigned 1 point for each source. Overall, higher scores indicated higher levels of personal social support. A total score of <20 signifies less social support, a total score of 20–30 signifies general social support, and a total score of more than 30 signifies satisfactory social support. In this study, the scale's Cronbach's alpha value was 0.940.

#### 2.2.4. Connor-Davidson Resilience Scale

The Connor-Davidson Resilience Scale (CD-RISC), developed by Connor and Davidson (17), consists of 25 items that were assessed using a five-point Likert scale ranging from 0 to 4: incorrect, rarely correct, sometimes correct, usually correct, and correct. Higher scores indicated higher levels of resilience (39). The questionnaire was divided into three dimensions: tenacity, strength and optimism (40). In this study, the CD-RISC overall Cronbach's alpha value was 0.962.

#### 2.2.5. Maslach Burnout Inventory-General Survey

We adopt the revised Chinese version of Maslach Burnout Inventory-General Survey (MBI-GS) (41, 42). The MBI-GS (Chinese version) consists of three subscales: emotional exhaustion (five items), cynicism (four items) and reduced personal accomplishment (six items). The scale was evaluated using a seven-point Likert scoring method, with scores from 0 to 6 indicating the frequency of their own feelings. For emotional exhaustion and cynicism, higher scores were associated with high levels of burnout. In the case of reduced personal accomplishment, higher scores indicated lower levels of burnout. The total Cronbach's alpha value of the burnout scale was 0.846, and the Cronbach's alpha values of the three subscales were 0.949, 0.945, and 0.937, respectively.

### 2.3. Statistical analysis

SPSS version 26.0 (IBM SPSS Statistics, USA) was used for data sorting and analysis. The measurement data are expressed as means ± standard deviations. For the basic situation of the data, a chi-square test was performed to assess the balance of grouping. If the data of both groups were normally distributed, a t-test was carried out for inter-group comparison; otherwise, the Mann-Whitney test was applied. For more than two groups, if the data satisfied a normal distribution and the variance was homogeneous, a one-way analysis of variance (ANOVA) was performed for inter-group comparison, and the least significant difference procedure was carried out for *post-hoc* comparison; otherwise, the data were analyzed by the Kruskal-Wallis test. The Spearman rank correlation method was used to analyze the correlation between social support, psychological resilience, burnout, stress, anxiety and depression. SPSS 26.0 (IBM SPSS Statistics, USA) software and its process program were used to analyze the intermediary effect, and the bootstrap method with deviation correction was used to test the regression coefficient to obtain the 95% deviation-corrected confidence interval. The confidence interval did not contain zero, indicating that the effect was statistically significant. For the homogeneity of variance test, the test level was 0.10, whereas the test level of the others were set at 0.05; that is, *p* < 0.05 indicated a statistically significant difference.

## 3. Results

### 3.1. Common method bias test

Exploratory factor analysis was performed on all items of the four scales using Harman's one-factor test for common method bias (43). The results showed that the explanation rate of the first principal component variation extracted by unrotated factors was 28.20%, which was less than the critical value standard of 40% (44), and there were 16 factors with eigenvalues >1. It was inferred that homologous variance was not the main reason for the co-variation among the variables. The synthetic reliability, the Omega coefficient, was also calculated (45, 46). The Omega coefficient used in this questionnaire was above 0.834, which performed well, and further data analysis was carried out.

### 3.2. General characteristics of the study sample

A total of 138 pediatric residents were included in the data analysis, of which the oldest was 36 years old and the youngest 22 years old. The average age was (26.25 ± 2.61) years old. See Table 1 for socio-demographic characteristics.

**Table 1:** Socio-demographic characteristics of the respondents.

		**Frequency**	**Percentage**
Total		138	
Gender	Male	39	28.3%
	Female	99	71.7%
Education level	PhD or MD	12	8.7%
	Master	38	37.5%
	Bachelor and others	88	63.8%
Working years	1 year	52	37.7%
	2 years	35	25.3%
	3 years	51	37.0%
Annual income	<¥50,000	53	38.4%
	¥50,000 –¥100,000	55	39.9%
	>¥100,000	30	21.7%
Category	Formal staff	22	16.0%
	Professional master student	42	30.4%
	Others	74	53.6%
Hometown	Urban	52	37.7%
	Rural	86	62.3%
Qualification	No physician qualification certificate	57	41.3%
	Have physician qualification certificate	81	58.7%
Clinical practice area	Pediatric internal medicine	118	85.5%
	Pediatric surgery	13	9.4%
	Others	7	5.1%

### 3.3. The status of negative emotions, social supports, psychological resilience, and burnout among pediatric residents

Analysis of the DASS-21 showed that the average scores of the subscales of stress, anxiety and depression were (9.83 ± 7.93), (8.26 ± 7.37), and (8.17 ± 7.77), respectively. The abnormal rate of stress was 18.8% (26/138), of which very severe stress, severe stress, moderate stress and mild stress accounted for 0.7% (1/138), 5.1% (7/138), 5.1% (7/138), and 7.9% (11/138), respectively. The incidence of anxiety was 47.8% (66/138), of which very severe anxiety, severe anxiety, moderate anxiety, and mild anxiety accounted for 7.9% (11/138), 7.9% (11/138), 22.9% (30/138), and 10.1% (14/138), respectively. The incidence of depression was 47.8% (67/138), of which very severe depression, severe depression, moderate depression, and mild depression accounted for 3.6% (5/138), 2.2% (3/138), 18.8% (26/138), and 20.3% (28/138), respectively. See Table 2 for details.

**Table 2:** Negative emotions of respondents.

		**Stress**			**Anxiety**			**Depression**		
		**Scores**	**Z**	***p***	**Scores**	**Z**	***p***	**Scores**	**Z**	***p***
**Total**		9.83 ± 7.93			8.26 ± 7.37			8.17 ± 7.77		
Gender	Male (*n* = 39)	11.79 ± 7.70	–2.065	0.039^*^	9.54 ± 7.01	–1.590	0.112	10.05 ± 7.68	–2.110	0.035^*^
	Female (*n* = 99)	9.05 ± 7.93			7.76 ± 7.48			7.43 ± 7.71		
Age	≤25 years old (*n* = 61)	9.70 ± 7.72	–0.028	0.978	8.26 ± 7.32	–0.026	0.979	7.87 ± 7.23	–0.082	0.934
	>25 years old (*n* = 77)	9.92 ± 8.15			8.26 ± 7.46			8.42 ± 8.21		
Education level	PhD or MD (*n* = 12)	8.00 ± 6.98	0.652	0.722	4.67 ± 4.70	3.835	0.147	5.17 ± 5.08	2.658	0.265
	Master (*n* = 38)	9.58 ± 8.80			8.42 ± 8.68			7.84 ± 8.77		
	Bachelor and others (*n* = 88)	10.18 ± 7.71			8.68 ± 6.98			8.73 ± 7.58		
Working years	1 year (*n* = 52)	9.69 ± 7.90	2.839	0.242	7.35 ± 7.08	2.971	0.226	7.50 ± 7.31	2.345	0.310
	2 years (*n* = 35)	11.14 ± 7.03			9.60 ± 6.54			9.60 ± 7.59		
	3 years (*n* = 51)	9.06 ± 8.56			8.27 ± 8.15			7.88 ± 8.35		
Annual income	<¥50,000 (*n* = 53)	11.74 ± 9.46	3.049	0.218	9.81 ± 8.76	2.273	0.321	9.70 ± 9.50	1.458	0.482
	¥50,000–¥100,000 (*n* = 55)	8.58 ± 6.49			7.49 ± 7.47			7.31 ± 5.99		
	>¥100,000 (*n* = 30)	8.73 ± 6.90			6.93 ± 5.80			7.07 ± 7.04		
Category	Formal staff (*n* = 22)	7.64 ± 5.91	4.975	0.083	4.64 ± 4.42	8.851	0.012^*^	4.91 ± 4.85	5.049	0.080
	Professional master student (*n* = 42)	12.52 ± 9.53			10.86 ± 9.17			10.24 ± 9.64		
	Others (*n* = 74)	8.95 ± 7.12			7.86 ± 6.41			7.97 ± 6.97		
Hometown	Urban (*n* = 52)	9.12 ± 7.46	–0.652	0.514	7.23 ± 6.46	–1.137	0.256	7.42 ± 7.10	–0.685	0.493
	Rural (*n* = 86)	10.26 ± 8.22			8.88 ± 7.84			8.63 ± 8.15		
Qualification	No physician qualification certificate (*n* = 57)	10.00 ± 7.92	–0.165	0.869	7.79 ± 7.02	–0.579	0.563	7.82 ± 7.53	–0.412	0.680
	Have physician qualification certificate (*n* = 81)	9.70 ± 7.99			8.59 ± 7.63			8.42 ± 7.97		
Clinical practice area	Pediatric internal medicine (*n* = 118)	10.19 ± 7.91	3.304	0.192	8.56 ± 7.40	1.984	0.371	8.56 ± 7.80	2.926	0.232
	Pediatric surgery (*n* = 13)	9.23 ± 8.43			6.77 ± 7.90			6.46 ± 7.97		
	Others (*n* = 7)	4.86 ± 6.41			6.00 ± 6.00			4.86 ± 6.41		

* *p* < 0.05.

The lowest SSRS score was 17, the highest was 57, and the average score was (32.21 ± 6.83). Among them, two respondents (1.4%) reported that they had received little social support, 53 (38.5%) had general social support and 83 (60.1%) had satisfactory social support. The objective support score was (5.92 ± 2.63), the subjective support score was (19.03 ± 4.282), and the support utilization score was (7.26 ± 1.86). The average score of the CD-RISC was (58.26 ± 15.66), including tenacity (42.56 ± 8.74), strength (28.54 ± 5.32) and optimism (13.16 ± 2.47). The overall average score of MBI-GS was (50.87 ± 20.59), of which emotional exhaustion accounted for (15.88 ± 7.11), cynicism (7.89 ± 5.50), and reduced personal accomplishment (20.62 ± 7.53). The incidence of burnout among pediatric residents was 51.4% (71/138), of which the incidences of severe burnout, moderate burnout, and mild burnout were 2.2% (3/138), 9.4% (13/138), and 39.8% (55/138), respectively.

### 3.4. Correlation analysis and mediating effect analysis

Stress among pediatric residents was negatively correlated with social support (*r* = −0.260, *p* < 0.01) as well as psychological resilience (*r* = −0.542, *p* < 0.01), but positively correlated with burnout (*r* = 0.488, *p* < 0.01); their anxiety was negatively correlated with social support (*r* = −0.265, *p* < 0.01) and psychological resilience (*r* = −0.566, *p* < 0.01), whereas positively correlated with burnout (*r* = 0.502, *p* < 0.01); depression, similarly, was negatively correlated with social support (*r* = −0.316, *p* < 0.01) and psychological resilience (*r* = −0.574, *p* < 0.01), but positively correlated with burnout (*r* = 0.528, *p* < 0.01). The correlation analysis of social support, psychological resilience, burnout, stress, anxiety and depression is shown in Table 3, indicating that H1 was verified.

**Table 3:** Correlation analysis.

**Variables**	**1**	**2**	**3**	**4**	**5**	**6**	**7**	**8**	**9**	**10**	**11**	**12**	**13**	**14**	**15**
**1. Social support**	1														
2. Objective support	0.705^**^	1													
3. Subjective support	0.881^**^	0.426^**^	1												
4. Utilization of support	0.575^**^	0.313^**^	0.285^**^	1											
**5. Psychological resilience**	0.430^**^	0.164	0.420^**^	0.386^**^	1										
6. Optimism	0.427^**^	0.221^**^	0.410^**^	0.309^**^	0.803^**^	1									
7. Strength	0.398^**^	0.142	0.400^**^	0.348^**^	0.971^**^	0.704^**^	1								
8. Tenacity	0.409^**^	0.160	0.390^**^	0.402^**^	0.954^**^	0.737^**^	0.885^**^	1							
**9. Burnout**	−0.341^**^	−0.211^*^	−0.280^**^	−0.356^**^	−0.584^**^	−0.377^**^	−0.565^**^	−0.619^**^	1						
10. Emotional exhaustion	−0.227^**^	−0.162	−0.233^**^	−0.075	−0.358^**^	−0.163	−0.374^**^	−0.356^**^	0.756^**^	1					
11. Cynicism	−0.267^**^	−0.170^*^	−0.193^*^	−0.286^**^	−0.417^**^	−0.218^*^	−0.405^**^	−0.463^**^	0.846^**^	0.736^**^	1				
12. Reduced personal accomplishment	−0.285^**^	−0.131	−0.218^*^	−0.460^**^	−0.581^**^	−0.502^**^	−0.535^**^	−0.608^**^	0.640^**^	0.093	0.314^**^	1			
**13. Stress**	−0.260^**^	−0.116	−0.184^*^	−0.282^**^	−0.524^**^	−0.341^**^	−0.542^**^	−0.486^**^	0.488^**^	0.464^**^	0.495^**^	0.255^**^	1		
**14. Anxiety**	−0.265^**^	−0.134	−0.176^*^	−0.333^**^	−0.566^**^	−0.395^**^	−0.564^**^	−0.540^**^	0.502^**^	0.444^**^	0.434^**^	0.320^**^	0.857^**^	1	
**15. Depression**	−0.316^**^	−0.178^*^	−0.233^**^	−0.358^**^	−0.574^**^	−0.407^**^	−0.576^**^	−0.544^**^	0.528^**^	0.423^**^	0.504^**^	0.355^**^	0.847^**^	0.858^**^	1

* *p* < 0.05;

** *p* < 0.01.

**Figure 1:**
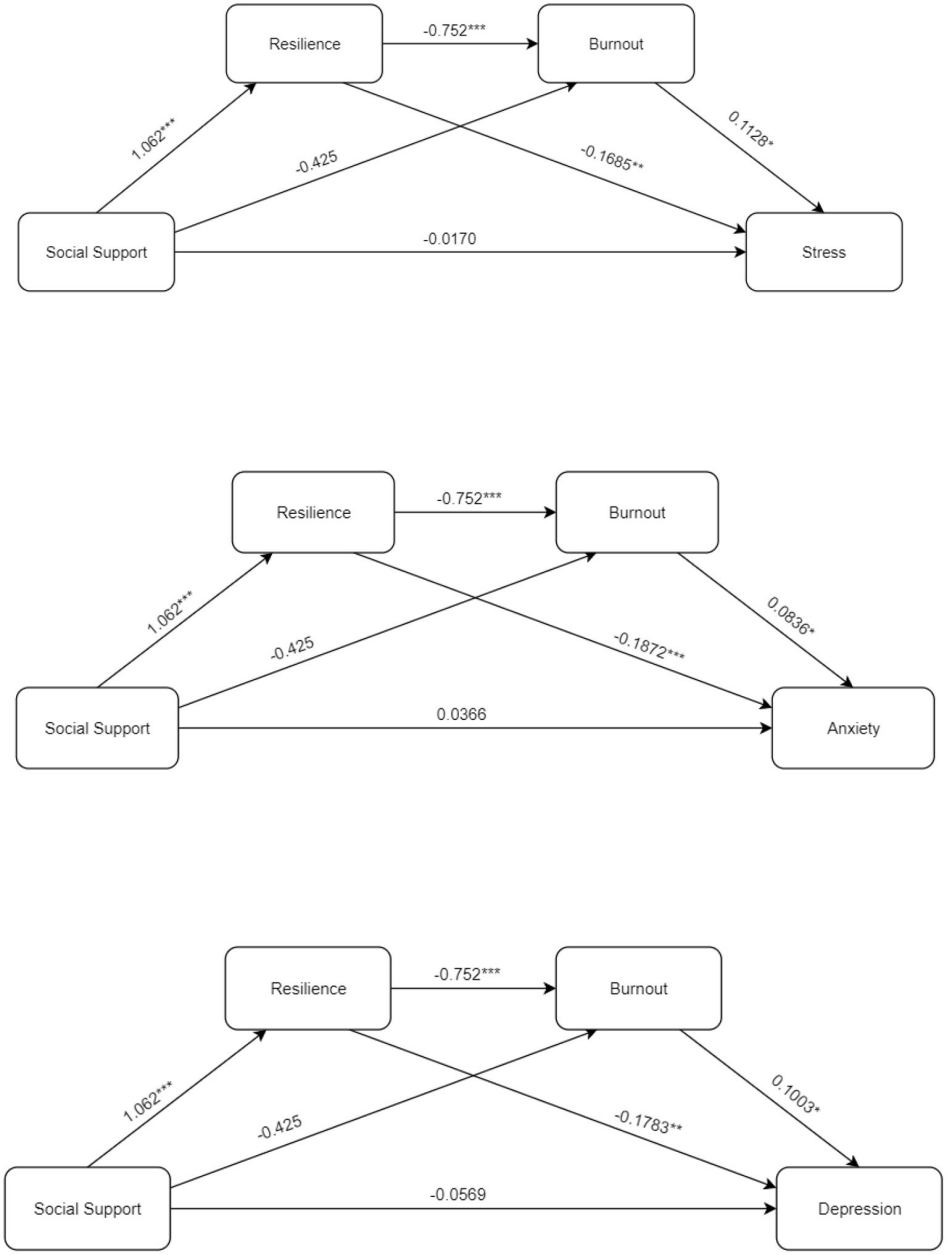
Beta values from the mediating effect analysis (***p < 0.001; **p < 0.01; *p < 0.05).

Taking the levels of stress, anxiety, and depression as dependent variables; social supports as independent variables, and psychological resilience and burnout as mediating independent variables; a chain-mediating effect analysis was carried out. The beta values obtained from the analysis are shown in Figure 1. Social support positively predicted resilience (β = 1.062, *p* < 0.001); psychological resilience negatively predicted burnout (β = 0.752, *p* < 0.001), stress (β = −0.1684, *p* < 0.001), anxiety (β = −0.1872, *p* < 0.001) and depression (β = −0.1783, *p* < 0.001); while burnout positively predicted stress (β = 0.1128, *p* < 0.05), anxiety (β = 0.0836, *p* < 0.05) and depression (β = 0.1003, *p* < 0.05).

It can be seen from Table 4 that the mediating effect of resilience between social supports and negative emotions was significant, indicating that H2 was verified; the mediating effect of burnout was not significantly associated with social supports and negative emotions, indicating that H3 was not verified; the chain-mediating effect of resilience and burnout was significantly associated with social supports and negative emotions, and it was a complete mediating role, which verified H4.

**Table 4:** Bootstrap analysis of mediating effect test between social supports and negative emotions.

	**Effect size**	**Boot SE**	**95% Bootstrap CI**
**Stress**			
Total effect	−0.3339	0.0954	(−0.5225, −0.1452)
Direct effect	−0.0170	0.0940	(−0.2029, 0.1690)
Total indirect effect	−0.3169	0.0690	(−0.4603, −0.1886)
Social support → Resilience → Stress	−0.1788	0.0586	(−0.3069, −0.0756)
Social support → Burnout → Stress	−0.0480	0.0281	(−0.1089, 0.0000)
Social support → Resilience → Burnout → Stress	−0.0901	0.0332	(−0.1597, −0.0309)
**Anxiety**			
Total effect	−0.2645	0.0897	(−0.4418, −0.0871)
Direct effect	0.0366	0.0883	(−0.1381, 0.2112)
Total indirect effect	−0.3010	0.0649	(−0.4329, −0.1839)
Social support → Resilience → Anxiety	−0.1987	0.0564	(−0.3204, −0.0987)
Social support → Burnout → Anxiety	−0.0355	0.0233	(−0.0869, 0.0017)
Social support → Resilience → Burnout → Anxiety	−0.0668	0.0298	(−0.1304, −0.0136)
**Depression**			
Total effect	−0.3689	0.0923	(−0.5514, −0.1864)
Direct effect	−0.0569	0.0905	(−0.2359, 0.1220)
Total indirect effect	−0.3120	0.0696	(−0.4579, −0.1856)
Social support → Resilience → Depression	−0.1892	0.0565	(−0.3146, −0.0918)
Social support → Burnout → Depression	−0.0426	0.0290	(−0.1102, 0.0007)
Social support → Resilience → Burnout → Depression	−0.0801	0.0339	(−0.1522, −0.0199)

## 4. Discussion

The abnormal rate of stress among the 138 pediatric residents in this study was 18.8%, and nearly half developed anxiety and depression. The residency period is the initial stage at which medical professionals embark on their formal career path, and stress may be related to excessive medical disputes and patients' high expectations (25). Tense doctor-patient relationships have a significant effect on mental health, job satisfaction, burnout and rates of drop-out among physicians (47, 48). In addition to intense clinical work, the income of residents is generally lower, more than 70% of pediatric residents in this study earning < ¥100,000 per year. Residency trainees also include many highly educated medical graduates. Some of these doctors may be over 30 years old when they start their training and may also be responsible for supporting their parents and other family expenses. Therefore, their financial pressure cannot be ignored, and improving the treatment of residents is still something that needs to be addressed at this time.

At present, it is believed that longer working hours are associated with greater work pressures, which may contribute to negative emotions (49). Some scholars constructed an effort-reward-imbalance model of occupational stress (50), which can be used to explain the occurrence of negative emotions. Negative emotions are not only detrimental to the mental health of physicians, but may also increase the incidence of medical errors (51). Screening out physicians with psychological abnormalities and encouraging them to seek help and treatment through educational actions may be a feasible way to help medical staff to reduce negative emotions (52). Psychological interventions had a positive and significant effect on negative emotions (53). The Vila Sana Program, which was established by the Norwegian Medical Association to provide free individual and group counseling services for all doctors in the country, improved the mental health of clinicians (54). There is also an urgent need in China to establish a psychological support service system for pediatric residents and for all physicians, to improve their mental health.

The results of this study showed that negative emotions among pediatric residents were significantly inversely associated with social support. Previous studies suggested that social support had preventive and regulatory effects on stress and depressive symptoms (55, 56), and social support can help residents to deal with stressors more effectively (57), which may be attributed to the way in which social support can shape brain activity (58). As seen in Table 3, male residents exhibited higher levels of stress and were more likely to suffer from depression. This suggested that the mental health of male pediatric residents should not be neglected. In China, female pediatricians far outnumber male pediatricians, which was also confirmed in this study. It was observed on a daily basis that female pediatricians are more likely to form partnerships and support each other, whereas Chinese men were less good at expressing emotions in traditional culture, which results in emotional problems. We also found that professional master's students are a relatively special group of residents, and anxiety is more obvious in this group, which might also be related to the current professional master's training model in China. Unlike other residents who only need clinical rotations to improve clinical theory and practice, professional master's students are required to engage in scientific research in addition to clinical work, which increases the risk of negative emotions. Considering the points outlined above, government authorities should reform the training of professional master's students.

Our study found that psychological resilience was one of the most important factors that mediated the relationship between social support and negative emotions, and that negative emotions among pediatric residents were significantly and negatively correlated with psychological resilience, similar to previous findings in other populations (35). Individuals with higher levels of psychological resilience were highly adaptable when confronted with adversities and work-related pressures, and they also demonstrated an ability to make decisions that were most favorable in the current situation, which enhances subjective wellbeing and quality of life (22). We also found that burnout was also an important factor in mediating the relationship between resilience and negative emotions. This is consistent with the conservation of resources theory (59) which holds that burnout arises from an imbalance between an employee's perceived levels of job investment and job reward. Moreover, burnout can cause employees to negatively evaluate themselves and others, and can also cause negative reactions. Therefore, in addition to increasing social supports for pediatric residents, it is also necessary to strengthen psychological resilience and reduce burnout, thereby alleviating negative emotions.

Some studies have explored how psychological resilience can be enhanced among doctors, and the most mainstream method involves providing mindfulness training (60–62). Before and after the implementation of some intervention measures, an improvement has been observed in resilience test scores (63, 64), although not all physicians showed a significant improvement, and some even exhibited decreased levels of resilience. However, these physicians nonetheless felt that similar interventions could encourage better peer relationships and play a positive role in strengthening resilience (61). Mindfulness-based interventions tend to focus on building resilience at the individual level, which is now believed to be determined by a combination of internal and external factors rather than a single intrinsic factor (33, 65), as confirmed by this study, and social support, as an external factor, can directly affect psychological resilience. Therefore, the results of the current study indicate that external supports, such as family support and peer support, play an equally important role in promoting psychological resilience. Some scholars have started to examine how external environments, such as organizations, affects individual resilience, proposing that it is possible to improve an individual's levels of resilience within an organization by addressing organizational-level issues (66). In China, literature that focuses on improving the psychological resilience of medical staff is scarce. As such, we need to learn from relevant foreign studies and develop psychological resilience intervention programs targeted at Chinese pediatric residents.

This paper presented a preliminary study on the current situation and multiple mediating paths of negative emotions among pediatric residents in China, and highlighted the role of social supports, psychological resilience and burnout in generating negative emotions. We call on the Chinese society to pay attention to the negative emotions of Chinese pediatric residents. Three ways to reduce negative emotions by strengthening social support, improving resilience, and alleviating burnout could be considered, which provides a basis for governmental policies on pediatric residents' occupational health. In the future, we can continue to study the factors affecting the negative emotions at different levels, such as individual, organization and society. The clinical intervention trial will be designed to explore the psychological intervention to improve the mental health of Chinese pediatricians.

This study had some limitations. The bootstrap analysis of the mediating effect of social supports and anxiety revealed that the total beta effect was smaller than the total indirect beta effect, although there may have been a masking effect. Therefore, future studies should increase the sample size to further examine the relationship between social supports and anxiety. Some of the conclusions presented in this paper were largely consistent with existing studies involving other populations, but further intervention studies should be carried out among the Chinese pediatric resident population to verify the findings. Self-report scales were used, and the results may have been impacted by social desirability bias.

## 5. Conclusion

Psychological resilience and burnout played a chain-mediating role between social supports and negative emotions. The whole society and government departments should pay attention to the emotional problems of pediatric residents, especially professional master's students and male residents. Strengthening social support, increasing psychological resilience, and relieving burnout may be the ways to reduce negative emotions. There is an urgent need to establish a psychological support service system to improve the mental health of pediatric residents. In conclusion, measures should be taken to improve the mental health of Chinese pediatric residents.

## Data availability statement

The original contributions presented in the study are included in the article/supplementary material, further inquiries can be directed to the corresponding authors.

## Ethics statement

This study was approved by the Ethics Committee of the children's hospital, Zhejiang University school of medicine (No. 2022-IRB-107). Written informed consent was obtained from the individual(s) for the publication of any potentially identifiable images or data included in this article.

## Author contributions

CS recruited participants, collected questionnaires, and wrote the draft. X-TD and J-HM performed the statistical analysis. Y-XH conceived the study and revised the draft. J-HM contributed to recruiting the participants and collecting the questionnaires. J-HM and WZ participated in the revision of the paper. All authors contributed to the article and approved the submitted version.
